# Interoceptive Ability and Emotion Regulation in Mind–Body Interventions: An Integrative Review

**DOI:** 10.3390/bs14111107

**Published:** 2024-11-18

**Authors:** Alessandro Lazzarelli, Francesca Scafuto, Cristiano Crescentini, Alessio Matiz, Graziella Orrù, Rebecca Ciacchini, Gaspare Alfì, Angelo Gemignani, Ciro Conversano

**Affiliations:** 1 Department of Civilizations and Forms of Knowledge, University of Pisa, 56126 Pisa, Italy; 2 Department of Languages and Literatures, Communication, Education and Society, University of Udine, 33100 Udine, Italy; francesca.scafuto80@gmail.com (F.S.); cristiano.crescentini@uniud.it (C.C.); alessio.matiz@uniud.it (A.M.); 3 Department of Surgical, Medical and Molecular Pathology and Critical Care Medicine, University of Pisa, 56126 Pisa, Italy; graziella.orru@unipi.it (G.O.); rebecca.ciacchini@med.unipi.it (R.C.); alfigaspare@gmail.com (G.A.); angelo.gemignani@unipi.it (A.G.); ciro.conversano@unipi.it (C.C.); 4 School of Advanced Studies, University of Camerino, 62032 Camerino, Italy

**Keywords:** interoceptive ability, emotion regulation, mind–body interventions, mindfulness, emotional expression

## Abstract

It is increasingly recognized that interoceptive ability, the capacity to detect, interpret, and consciously integrate signals related to the physiological condition of the body, is central to emotion experience and regulation. Interoceptive ability can be trained and improved through mind–body interventions. This article attempts to provide an integrative review of the link between interoceptive ability and emotion regulation in mind–body interventions. To this aim, (1) we address the constructs of interoceptive ability and mind–body interventions in relation to the double pathway of emotion regulation, and (2) we include a review of selected empirical and qualitative studies. These show that mindfulness meditation affects the brain–body axis through top-down processing, improving both interoceptive ability and emotion regulation. Interventions based on bottom-up processing through body movement and emotional expression are illustrated, but it is argued that they are still under-investigated. In light of the literature reviewed, we contend that interoceptive ability is a crucial aspect associated with the effects of mind–body interventions on emotion regulation. Additionally, we suggest that if studied through both quantitative and qualitative methods, interoceptive ability may serve as a general construct that allows a more integrated view of the polarities related to the spectrum of embodied experience: top-down and bottom-up emotion processing, observational and non-observational body awareness, conscious and unconscious level of interoception.

## 1. Introduction

The last decades have witnessed a surge of interest in interoception, firstly introduced by Sherrington [1] to account for the signals originating from within the visceral tissues. Research on interoception found new grounding in neuroimaging studies, when Craig [2] first pointed out the neural correlates involved in the representation of the afferent body signals in the central nervous system (CNS). Craig finely illustrated that at the level of brain processing, once the peripheral information is integrated in the mid-insula, the CNS forms a higher order representation of the body status, which gives rise to “the sense of the physiological condition of the body” [2] (p. 655). The insula receives input from the homeostatic pathways [3], the somatosensory cortex, and from other sensory channels such as the visual, auditory, and vestibular. Moreover, it integrates information from the amygdala and hypothalamus [4]. Neuroimaging studies converge in considering high brain regions such as the insula, ACC, and somatosensory cortex central to the neural network involved in the representation of the internal state of the organism [2,5]. Among these, the anterior insular cortex (AIC) plays a crucial role in the re-representation of interoceptive signals and is supposed to provide the neural substrates of subjective awareness [6].

Partly informed by neuroimaging studies, partly drawing on a better comprehension of the brain–body axis, interoception has been variously defined as: “the afferent information that arises from anywhere and everywhere within the body, not just the visceral organs” [7]; “the multi-sensory, multimodal integrated percept of the body state” [8]; “the processes by which an organism senses, interprets, integrates, and regulates signals from within itself” [9]; “the overall process of how the nervous system (central and autonomic) senses, interprets, and integrates signals originating from within the body, providing a moment-by-moment mapping of the internal landscape of the body across conscious and nonconscious levels” [10]. These definitions extend the concept of interoception to include brain–body processes along a continuum that goes “from the physical responses in body and brain representation up to (and beyond) interoceptive metacognitive insight and conscious awareness” [11].

However, discrepancies within this research field have been highlighted together with the need to further refine the current conceptualization of interoception and its dimensions [12]. In particular, it has been argued that including both the processing of internal body stimuli by the nervous system (phenomenon-based definition) and homeostatic pathways (physiological definition) under the definition of interoception is problematic, because the processing of internal states also involves non-homeostatic pathways like those generally assigned to exteroception [12]. For this reason, Desmedt et al. [12] (p. 33) proposed a broader definition that “includes the top-down and bottom-up processes by which an organism senses, interprets, and integrates signals from within itself and below the skin, across conscious and nonconscious levels”.

In this article, we acknowledge the inconsistencies abovementioned and draw on the distinction between the afferent homeostatic pathways (from receptor site up to primary areas of processing) and interoception just as the result of higher order processing [8]. We mainly focus on the second dimension, namely interoception as an integrated percept. Given that interoceptive perception can be improved through mind–body processes, we take interoceptive ability as an appropriate theoretical construct.

It is increasingly recognized that interoceptive ability is relevant to emotion by supporting the detection, interpretation, and integration of early body reactions in response to emotional stimuli [13]. This aligns with peripheral theories of emotion, according to which the capacity to detect physiological changes to internal or external events allows to more effectively identify, comprehend, and modulate emotional reactions [14,15,16], even during critical situations or clinical conditions (i.e., [17,18,19]). Therefore, the perception of internal body states is intrinsically related to emotion experience and regulation. Interoceptive ability has also been proposed as the foundation of affective consciousness or affective-homeostatic basic consciousness [20], a concept that comes close to the *core self* [21], *protoself* [15], and *minimal self* [22]. In other words, consciousness originates from affective states and interoceptive ability informs the organism about the physiological changes in its body’s internal milieu from homeostasis to affect [20]. This implies two modalities of body awareness: observational awareness, which involves attending to the body as an object, and non-observational awareness, where the body is given as the subject of experience and does not involve intentional attention [23]. It is assumed that these modalities correspond, respectively, to the conscious and subconscious level of interoception.

Interoceptive ability and emotion regulation can be trained and improved through mind–body interventions. With the latter, we refer to a group of practices that emphasizes using the mind or brain in conjunction with the body by initiating from mental processing at the level of the cerebral cortex (top-down) or from afferent pathways from the periphery to the brainstem and cerebral cortex (bottom-up) [24]. Whereas interventions such as hypnosis, meditation, and contemplative practices refer to intentional mental activities that work primarily top-down, interventions that facilitate spontaneous body movement and emotional expression rely mainly on peripheral sensory afferents that work bottom-up.

Because these bidirectional processes also define ways of experiencing one’s body and emotions, exploring the interplay between interoceptive ability and emotion regulation in this context becomes a prominent research subject from a clinical point of view. Indeed, this article calls attention to such linkage and suggests that the two constructs cannot be separated in studying the effects of mind–body interventions. Given the complex and broad theoretical and practical implications of this relationship, we opted for a narrative review. Unlike a systematic review, which focuses on a narrow question, a narrative review allows us to include a wide variety of studies and elaborate them, in order to explore potential links and gain new insights about a still under-investigated question. Specifically, we carried out an integrative review, with the intention to combine different methodologies (e.g., RCT, clinical trials, case studies) and include selected empirical studies and theoretical articles drawn from the bibliography of the former. We aim at exploring the role of interoceptive ability in mind–body interventions and its relationship with emotion regulation. To our knowledge, no other reviews studied this relationship, thus confirming the innovative value of this effort.

## 2. Interoceptive Ability

In light of the theoretical considerations presented in the introduction, we conceive interoceptive ability as the capacity to detect, interpret, and consciously integrate signals related to the physiological condition of the body. Therefore, we refer specifically to the interoceptive processes that occur at, or can access, the level of conscious awareness. Various operative frameworks have been proposed to address these processes. In the last decade, a prominent and widely accepted model emerged within interoception research [25,26], which articulates and measures interoceptive ability along three psychological dimensions: accuracy, sensibility, and awareness.

Interoceptive Accuracy (IAc), alternatively defined as sensitivity, refers to the ability to precisely and correctly monitor changes in internal events [10,26]. It aims at objectively quantifying individual differences in behavioral performances and therefore is considered as the core element of interoception. IAc is usually assessed using experimental tasks in which physiological events are objectively measured while participants report the body sensations they experience. Multiple tasks exist for different visceral axes, even though the heart–brain axis has been the largest focus in the study of IAc. The heartbeat counting task [27] and the heartbeat detection task [28] are the most utilized. In the first one, participants are asked to report the number of heartbeats felt during a certain time interval, and accuracy is measured on the difference between actual and reported heartbeats. In the second one, participants listen to sequences of auditory tones and report whether these are synchronized or delayed compared to their heartbeats, determining detection indices of sensitivity and bias. Yet, the construct validity of both tasks has been questioned [29]. Moreover, a weak correspondence between different visceral axes (e.g., measures of cardiac and respiratory IAc) has been reported [30,31]. These inconsistencies pose a limit to the possibility of reaching high validity in the objective measurement of interoceptive ability.

Interoceptive sensibility (IS) consists of self-report accounts of how internal sensations are experienced. This dimension indicates a subjective measure that reflects both individuals’ beliefs in their interoceptive ability and the confidence in their performance accuracy on an experimental task. IS may be assessed through either self-report questionnaires (i.e., the Body Perception Questionnaire—BPQ [32]; the Multidimensional Assessment of Interoceptive Awareness—MAIA [33]) or confidence ratings of how accurately an individual performs during a task of interoception [26]. Whereas BPQ is a questionnaire that assesses the frequency of specific body stress reactions in organs that are innervated by the autonomic nervous system (ANS), MAIA contains subscales that measure multiple aspects of IS, including the convergence between body states and emotions. Differently, confidence rating focuses on the perceived accuracy of response, using a continuous visual analog scale (VAS) that ranges from “no heartbeat awareness” to “full perception of heartbeat”.

Interoceptive awareness (IA) is conceptualized as a measure of metacognitive awareness of interoceptive ability. Whereas IA and IAc have been previously treated as synonymous and interchangeable, within this multidimensional framework IA refers to the correspondence between IAc and IS [26]. Yet, IA is widely used to point to the conscious and evaluative interpretation of internal body signals in the context of contemplative practices [34].

**Figure 1:**
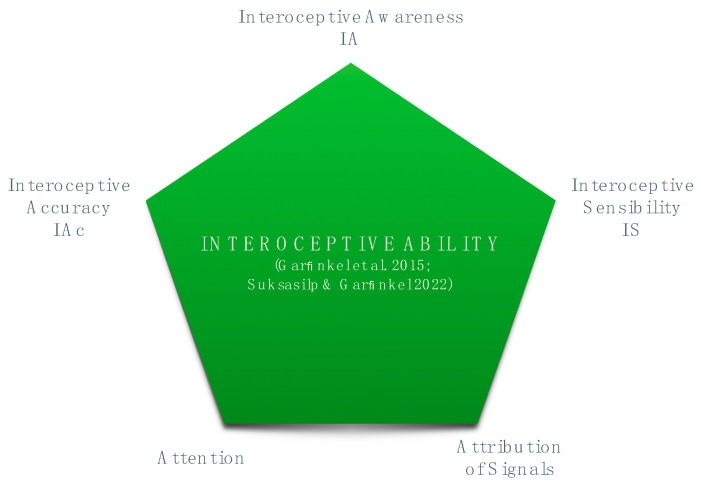
Integrative definition of Interoceptive Ability based on a Multidimensional Framework [26,31].

A recent contribution extends this model to include two additional dimensions, respectively, interoceptive attention and attribution of signals [31]. The first one refers to the mode of attention through which interoceptive signals reach conscious awareness: attention may amplify or attenuate internal body signals. The second one reflects the specific attribution and interpretation of interoceptive signals and may be influenced by the dispositional style, affect, and sociocultural context. These two dimensions are relevant to clinical conditions. For instance, whereas high attention to body sensations could cause hypervigilance, negative interpretation of interoceptive signals could foster catastrophizing [34]. For this purpose, MAIA is a widely used self-report questionnaire, specifically developed to differentiate mindful or resilience-enhancing from maladaptive interoceptive processes associated with hypochondriasis, somatization, and anxiety disorders [33,35] (for a comprehensive representation of the multidimensional framework see Table 1 and Figure 1).

Although the majority of the articles that we reviewed endorsed this multidimensional model, it is important to note that there is a lack of empirical convergence between the conceptualization of conscious interoceptive signals and the means used to measure them [12]. With regard to this discrepancy, Desmedt et al. [12] (p. 38) recommend the development of a more comprehensive and precise conceptualization of interoception whose model should include the following: (1) constructs at different level of specificity (have a hierarchical structure); and (2) more dimensions to cover all interoceptive phenomena. In particular, the authors illustrate a framework in which interoception can be hierarchically conceptualized according to the level of specificity of constructs (level of psychological or physiological processing). In this framework, interoceptive ability covers broad factors: (1) interoceptive attention; (2) interoceptive sensing; (3) interoceptive interpretation; and (4) interoceptive memory.

Finally, because interoceptive processing has been proposed as the very foundation of affective consciousness [20], it is important to also consider the phenomenology of interoceptive ability beyond the constructs listed above. From a first-person perspective, interoceptive ability is linked not only to the awareness of the body as the object of experience and reflective thought, but also to the bodily background feeling, the ultimate primitive of consciousness [20]. Interoceptive and homeostatic processes characterize this fundamental affective layer, “informing the subject about the overall state of her internal milieu and thereby contributing to the maintenance of the overall physiological balance within the organism” [20] (p. 27). In this regard, phenomenological methods, which investigate how the subject makes sense of her own lived experience in the world, can help us to better understand how interoceptive ability shapes consciousness at both the pre-reflective and attentional level of embodied awareness, also known as observational and non-observational awareness [23]. For instance, in a phenomenological interview, the participants are asked to describe or choose the scenes that induced certain feelings. The interview focuses on the experience associated with the selected scene assuming the phenomenological attitude of judgment suspension and the principle of evocation. Examples of questions include “what do you feel”, “how do you perceive it”, “how do you feel at a certain time” [36].

Other qualitative methods, such as in-depth interviews and observational data collection, could also be used for studies from a cross-cultural perspective. These qualitative methods may inform the interplay between cultural contexts and interoception, revealing possible disparities in reported bodily sensations and perceived physiological changes across diverse cultural groups. As a matter of fact, African Americans are more likely to report angina during treadmill exercises, greater pain after surgeries, and higher levels of chronic pain in medical conditions such as HIV/AIDS, glaucoma, and arthritis than their European American counterparts [37]. Distinct cultural variances are also reported concerning accounts of bodily symptoms linked to emotional distress. In contrast to the prevalent Western manifestation of anxiety that predominantly exhibits affective symptoms, anxiety in non-Western cultural contexts is related to distinct somatic experiences. For instance, Cambodian refugees may report neck pain and gastrointestinal issues, the Chinese community may associate anxiety with dizziness, African Americans may experience isolated sleep paralysis, and among West Africans, there might be reports of genital narrowing as expressions of anxiety [37]. This underlines the significant role that the intertwining of interoceptive ability and the socio-cultural framework plays in shaping the individual perception of the state of health.

### 2.1. Interoceptive Ability and Emotion

There is an increasing convergence in the relationship between interoception and emotion. This link is especially supported by empirical studies that show how interoceptive ability (mostly measured as IAc) is related to emotion experience and regulation (see Supplementary Table S1). Two main results emerge from the studies. On the one hand, data based on fMRI and EEG analysis show similar patterns of brain activity during both interoceptive tasks and emotion elicitation [5,38,39,40]. In line with Craig’s findings [2], the processing of visceral information in the right anterior insula lies behind the overlap between interoceptive and emotion perception. On the other, studies show how IAc is positively associated with a higher emotional awareness, intensity, and regulation [38,41,42,43,44,45,46,47]. In other words, there is a close association between the perception of visceral signals, the awareness of emotional states and their regulation. In addition, emotion regulation may be a key factor for well-being in mind–body interventions. Among mind–body interventions, the so-called psychosomatic mindfulness, for instance, showed an improvement in psychological well-being through the mediation of emotion regulation [48].

The link between interoceptive ability and emotion is gaining higher interest in the field of psychosomatics and psychopathology [47,49,50,51,52,53]. Understanding this connection could offer valuable insights into the psychological and emotional experiences of individuals living with chronic conditions [54] and potentially inform more targeted therapeutic approaches. In particular, it has been pointed out that positive changes in IAc induced by contemplative practices are associated with an improved capacity to verbalize emotional states and to a subsequent decrease in alexithymia [47]. On the contrary, low IS has been correlated to increased levels of alexithymia [51]. Similarly, altered interoceptive processing is associated with clinical conditions characterized by emotion dysregulation. For instance, reduced amplitudes of heart evoked potential (HEP), an indicator of cortical processing of afferent bodily signals, were revealed in patients with bipolar personality disorder [50], while a reduced IAc was reported in anorexia nervosa patients [52].

Beyond the clinical context, in the psychosocial field, IAc could play a relevant role in the change in social behaviors. Indeed, it moderates the extent to which bodily responses are related to cognitive–affective processes such as intuitive thinking and decision-making [55]. Hence, people with higher interoceptive ability are more prone to using body sensations as guides for decision-making.

In sum, these findings emphasize how interoceptive ability is central to emotion experience and regulation, but there is still a lack of research that investigates this relationship in the field of mind–body interventions. We will take this area into consideration in the next paragraph, but before that, it is important to address interoceptive ability according to a double pathway of emotion regulation.

### 2.2. Interoceptive Ability in the Double Pathway of Emotion Regulation

Emotion regulation refers to the ways by which individuals modulate automatically or intentionally their emotional experiences and expressions in everyday life [56]. In the psychotherapeutic context, two main approaches to emotion regulation have been highlighted: Cognitive Emotion Regulation Model (CER) and Experiential-Dynamic Emotion Regulation (EDER) [57]. The former is based on the appraisal theory of emotion [58,59]. The main tenet of this theory is that emotional experience is given by the appraisal (operated by high order mental processes) of physiological changes coming from the periphery. Therefore, emotional responses unfold over different phases (attention, cognitive appraisal, and behavior) along a temporal order. Within this framework, the process model differentiates emotion regulation strategies according to the time they intervene in the unfolding emotional response: we can distinguish between antecedent-focused strategies, which are used before the emotion response tendencies become active, and response-focused strategies, which are used once the response tendencies are generated [56,60,61].

Studies aimed at exploring the role IAc plays in emotion regulation (see Table S1) focused mainly on two specific strategies from the process model: cognitive reappraisal (antecedent-focused), interpreting a situation in a way that influences its emotional impact, and expressive suppression (response-focused), inhibiting the expression of emotional behavior [43,44,45,46]. Füstös et al. [43] pointed out a positive correlation between IAc and more successful emotion regulation abilities in response to negative affect. Specifically, they have shown that IAc facilitates the downregulation of affect-related arousal when applying reappraisal as emotion regulation strategy, therefore it moderates the above relationship [43]. Similarly, Weiss et al. [44] found that IAc supports self-regulation capacities and is positively correlated with frustration tolerance as well as with affect differentiation and affect tolerance. In addition, Kever et al. [45] found that IAc was not only positively correlated with reappraisal regulation strategy, but also with a more beneficial use of suppression strategies [45]. In line with these findings, Pollatos et al. [46] demonstrated that higher IAc is associated with more efficient emotion regulation strategies in reducing aversive effects provoked by social exclusion.

Although widely accepted and used in studies on emotion and interoception, this model is limited in addressing mind–body interventions focused on emotional expression. EDER has been proposed as an alternative framework to explain techniques that work on emotion regulation by allowing patients to express and act their emotions, and subsequently become aware of their body sensations [57]. This model draws on affective neuroscience [15,21,62], which stresses that emotions are generated in subcortical areas and are expressed through a sequence of actions appropriate to the nature and intensity of the stimulus. Primary emotions are prewired and partially independent of cognition since an emotional response, triggered by a particular stimulus, produces a coherent expression-action [57]. According to this view, emotions are not inherently dysregulated, but rather experiences that inform cognitive appraisal of the situation and that may be expressed into healthy actions [57]. Dysregulation is due to excessive conditioned anxiety or defense mechanisms that create defensive affects (for the assessment of defense mechanisms see [63,64,65]).

EDER also finds support in the enactive approach to emotion [66] and is validated by some empirical studies that show how the timing of emotion is variable and that stress the crucial function of the sensorimotor processing in generating emotional experience [67,68]. On the one hand, studies have shown that the inhibition of those facial muscles involved in emotional expression through Botox injection weakens the corresponding emotional experience [69] and reduces the activity of the amygdala and brainstem [70]. On the other, there is compelling evidence that the enactment of a facial expression or a posture facilitates the corresponding emotional experience and influences the way in which the emotional information is processed [67,69,71,72]. In line with this view, Tang et al. [73] emphasized the role of the body in the mindfulness field by using the term “bodyfulness”, which refers to the gentle adjustment and exercise of body posture with a full awareness, in order to achieve presence, balance, and mind–body coordination. This is consistent with the hypothesis, central to the embodiment framework, that information processing begins in the sensorimotor periphery [74,75]. This hypothesis postulates a close connection between the perceptual, soma-sensory, viscero-sensory, and motoric systems involved in the emotional experience. The main tenet of the embodied approach is that the state of the body, including posture, breathing, action, and muscle contractions are all relevant to emotional experience. An implication of the overlapping of the sensory and motor neural circuits, and their connection with subcortical and cortical brain regions, is that body movement and emotional expression may become a channel for experiencing and regulating one’s emotions.

In sum, research on interoception largely employed the CER approach, mainly rooted in the appraisal theory. Here, empirical evidence started to show how interoceptive ability (so far mainly studied through IAc, but extendable to other dimensions) facilitates emotion regulation based on cognitive reappraisal and expressive suppression strategies. Yet, the role of interoception in the EDER approach is still under-investigated. Particularly, little is known about interventions based on the enactment of the sensorimotor system through spontaneous body movement and emotional expression.

## 3. Mind–Body Interventions: Top-Down and Bottom-Up Processing

Mind–body interventions, also defined as mind–body therapy, refer to a variety of practices and techniques used for therapeutic or self-cultivation purposes, which encompass contemplative practices, breathwork, body movement, and emotional expression, among others. They can be used for therapeutic purposes in programs for the promotion of mental health and well-being, in body-oriented psychotherapies, or in a variety of ritualistic and spiritual traditions from a cross-cultural perspective. Therefore, the setting and sociocultural context in which mind–body interventions are applied is relevant to their operative definition. For the purpose of this article, we do not intend to examine each of them (for a general taxonomy of mind–body interventions and empirical evidence of their effectiveness see [76]), but rather to define the main criteria that distinguish these interventions in relation to interoceptive ability.

Mind–body practices influence the bidirectional communication pathways between the brain and body and take place via three routes: the autonomic nervous system (ANS), the endocrine system, and the immune system [77]. Here, we focus on the first one, as the psychophysiological processes involved in the ANS can reach conscious awareness once represented and integrated at the level of the CNS. In this regard, it has been hypothesized that a key mechanism of mind–body interventions is the interaction of brain processes, which take place in the CNS, with the signals coming from the periphery through the activity of the ANS [73]. Given the bidirectional communication between CNS and ANS, we can take into consideration the psychophysiological changes associated with the brain–body axis and draw a broad distinction between top-down and bottom-up processing.

Top-down processing takes place mainly via high-order processes of consciousness. It refers to conscious and intentional mental activities, even though unconscious processes also seem to be involved [24]. Psychophysiological changes initiated by top-down processing encompass, among others, the downregulation of body arousal, decreased expressive behavior, reduced emotional experience, and decreased sympathetic activation of the cardiovascular system. In other words, top-down processing uses mainly voluntary attention on the physiological condition of the body to monitor and attenuate signals coming from the subcortical regions, enhancing emotional awareness and the capacity to observe emotions in a non-engaging stance. Mind–body approaches based predominantly on top-down processing are contemplative practices with or without body movement. Relevant examples of those practices are the so-called mindfulness-based interventions such as Mindfulness Based Stress Reduction (MBSR; [78]), Mindfulness Based Cognitive Therapy (MBCT; [79]), and Mindful Awareness in Body-oriented Therapy (MABT), a therapeutic approach that trains interoceptive awareness skills to promote emotion regulation and well-being using the combination of mindfulness, self-massage, and psychoeducational approaches [80].

Tang et al. [73] pointed out two main changes associated with mindfulness training. At the level of the ANS, practitioners show lower chest respiratory rate, heart rate, and skin conductance response, but greater belly respiratory amplitude than the control group [81], suggesting greater parasympathetic regulation of ANS. At the level of the CNS, meditation activates the ACC, which is associated with heart frequency variability, and shapes a self-control system in the regulation of the ANS together with the amygdala and vagus nerve [73]. Therefore, an enhanced regulation of the ANS through the self-control system of the ACC probably reflects a better coordination between body and mind [73].

Bottom-up processing, on the other side, is initiated by somato-sensory, viscero-sensory, and chemo-sensory receptors that travel along afferent pathways from the periphery to the brainstem and cerebral cortex and influence high order mental processes [24]. Unlike the previous one, bottom-up processing is also characterized by the activation of body arousal, sympathetic activation of the cardiovascular system, increased expressive behavior, and increased emotional experience. Mind–body approaches that elicit bottom-up processing emphasize spontaneous body movement and emotional expression.

Given that these interventions may trigger highly intense emotional experiences outside voluntary cortical control, they are usually proposed within a therapeutic domain. For instance, catharsis, the experience of tension resolution or relief resulting from the release of highly intense emotions [82] is a well-known intervention in body-based psychotherapies. Although empirical research on catharsis is virtually inexistent, theories in line with the EDER model view catharsis as the completion of an emotional action sequence that would have occurred as a natural reaction, but that it was previously restrained or interrupted [83,84,85]. From this point of view, repressed affects are blocked actions, and catharsis may serve as a means of helping patients to resolve these blocks in a safe therapeutic setting, in order to “turn the patient’s attention to whatever emerges” [83] (p. 56). According to this embodied theory, we may hypothesize that emotional expression and catharsis enhance interoceptive ability by triggering involuntary attention on the unblocked feelings that were repressed.

To date, it is still not clear what is the role of interoceptive ability in bottom-up processing. In the field of body-oriented psychotherapy, Payne et al. [86] emphasize the interaction between the ANS and other three somatic systems/that are subcortical structures (i.e., emotional motor system EMS, reticular arousal system RAS, and the limbic system LS), which form the so-called “core response network” [86]. The construct “core response network” resembles “core self” [21], “protoself” [15], and “minimal self” [22]. According to the authors, interoceptive as well as proprioceptive signals are relevant to the outcome of the therapy, considering that the ANS and subcortical centers are not separate from the somato-visceral and musculoskeletal nervous systems. This model further explores the possibility that affective consciousness, which is related to subcortical regions and the core response network, is central to working on bottom-up emotion regulation.

**Figure 2:**
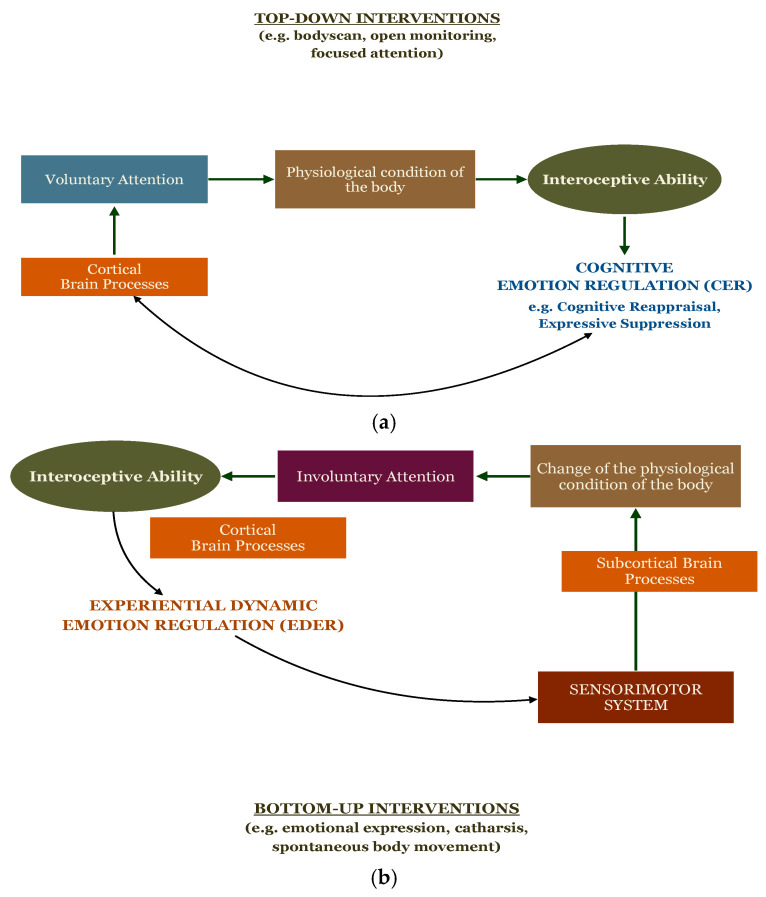
The Relationship between Voluntary and Involuntary Attention [87] and Top-Down/Bottom-Up Processing of Interoceptive Ability. Note: (**a**) High-order processes of consciousness direct voluntary attention on the physiological condition of the body. This, in turn, determines the grade and quality of interoceptive ability, which facilitates emotion regulation strategies based on the CER model (these involve high-order processes); (**b**) the enactment of the sensorimotor system through spontaneous body movement and emotional expression activates subcortical brain processes and produces a change in the physiological condition of the body. This, in turn, triggers involuntary attention to the body, leading to the conscious perception and elaboration of interoceptive signals. Interoceptive ability, through the interplay between cortical and subcortical brain processes, is central to experiential dynamic emotion regulation (EDER).

Since all body signals that access consciousness are ultimately represented and processed at the cortical level, we can differentiate between top-down and bottom-up processing according to the modality of consciousness and attention that is used to attend to those signals [87]. In the first one, high-order processes of consciousness are used to direct voluntary attention on the physiological condition of the body, which determines the grade and quality of interoceptive ability. In the second one, the enactment of the sensorimotor system through spontaneous body movement and emotional expression activates subcortical brain processes and produces a change in the physiological condition of the body. This, in turn, triggers involuntary attention to the body, leading to the conscious perception and elaboration of interoceptive signals (see Figure 2).

It is important to note that the top-down/bottom-up categorization is an oversimplification, as it is frequently the case that mind–body interventions actually involve a combination of both types of processing [24]. For instance, Eastern movement-based contemplative practices such as Qigong, Tai Chi and Yoga combine bottom-up pathways, activated by peripheral sensory afferents from the interoceptive and proprioceptive systems, and top-down pathways, activated by focused attention and the intention to relax. Among these, Qigong includes more bottom-up techniques in that it stresses movement and breathing, or even spontaneous and expressive movements under the form of Spontaneous Qigong and Daoist Qigong [88,89].

The overlapping of top-down and bottom-up processing has been pointed out in mindfulness-based practices as well [90]. For instance, a so-called psychosomatic mindfulness training [91] combines top-down processes of mindfulness meditation with bottom-up processes, including interoceptive exercises based on body movement and breathing. It focuses primarily on physical self-experience deriving from mind–body interventions and therapies that consider past experience as embodied in current physiological states and action tendencies [92]. The program has been developed in a variety of forms, not only as a means of health promotion, but also as a therapeutic intervention through techniques such as bodyscan, which facilitates the integration between body sensations, emotions, thoughts, and memories. It also uses relaxing and activating exercises to stimulate the parasympathetic and sympathetic systems, as well as emotional expression. To date, the health promotion program was verified to affect internalizing and externalizing problems in children [93] and in adolescents [94], as well as eudaimonic well-being in early adolescents [95].

Parallel to top-down and bottom-up processing, other criteria of classification for mind–body interventions have been proposed. For instance, Gibson [96] emphasized the type of attentional style (e.g., focused attention and open monitoring), which leads to different insights and understanding of interoceptive signals. Schmalzl [97] underlined the type of movement involved, which can be spontaneous and involuntary or controlled and voluntary, or even imagined movement involving interior sensations of heat, vibration, and energy currents. Tarsha et al. [76] elaborated a taxonomy based on three factors: movement/non-movement, the administration method (e.g., self-administered or delivered by another person), and physical contact (whether or not touch is utilized).

Furthermore, it is important to make a distinction between the contexts in which mind–body interventions are applied. Several of them are rooted in Eastern spiritual traditions and are practiced daily in both religious and secular ways. For instance, ethnographic studies of Qigong [89] and Buddhist meditation [98] show how mind–body techniques are central to healing rituals and self-cultivation paths for well-being in contemporary Chinese societies. In Western contexts, contemplative and mindfulness-based practices are nowadays offered also in daily recreational contexts and group programs aimed at promoting mental health. In addition, bottom-up interventions based predominantly on spontaneous body movement and emotional expression are integrated in body-psychotherapies [99] such as bioenergetic analysis [100], sensorimotor psychotherapy [92], and somatic experiencing [86]. Similarly, the so-called interoceptive exercises, which are used to sustain interoceptive sensations connected with problematic cognitions, are often integrated as part of exposure-based approaches in cognitive-behavioral treatments. Interoceptive interventions showed positive effects on fear and anxiety-related disorders (e.g., [101,102]), psychosomatic disorders such as irritable bowel syndrome [103,104], and phobias [105]. The exposure may also reduce fear of arousal-related sensations, due to the elicitation of trauma-relevant memories as in PTSD symptoms [106]. The psychotherapeutic setting is particularly important in developing interoceptive ability through bottom-up interventions, as the therapeutic relation allows to experience, elaborate, and integrate emotional states at both the pre-reflective and attentional level of consciousness. Hence, top-down and bottom-up interventions can also be differentiated according to the aforementioned criteria, as summarized in Table 2.

### Insights from Case Studies

Noticeable insights come from case-study reports related to interoceptive interventions (see Table S2), which can help us to better understand the application of interoceptive ability in a clinical context. For instance, a brief program of 11–13 sessions of interoceptive and in vivo exposure, combined with cognitive-behavioral treatment, reduced choking phobia and increased weight gain [107]. A case series study showed that four-sessions of Interoceptive Exposure (IE) exercises combined with traditional Cognitive Behavioral therapy (CBT) predicted a decrease in interoceptive deficits and anxiety in individuals with eating disorder [108]. Specific IE exercises were selected from stakeholder’s feedback and ideographically applied to every participant, in order to evoke the highest distress caused by one’s typical experiences. Hence, the intervention was aimed to increase breathing and heart rate, tension in the chest, dry mouth, dizziness, nausea, and other uncomfortable sensations associated with loss of control. Exercises included paying attention to sensations during and after; hyperventilation (engage in rapid and shallow breathing); thin straw breathing; spinning in place; gulping water until full; smelling high calorie food; wearing tight clothes. Between sessions, participants were asked to practice IE daily.

Similarly, in a case study of an adolescent with eating disorder, a six-sessions-exposure interoceptive program called acceptance-based interoceptive exposure (ABIE), was revealed to improve tolerance of disgust and to build counterconditioning to the interoceptive cues via explicit pairing of positive experiences (e.g., listening to favorite music) with feeding [109]. As a result, the adolescent reported a higher weight gain and reliable improvement in symptoms. This intervention was combined with the teaching of some mindfulness skills to resist the distress generated by interoceptive sensations (e.g., pressure in stomach, nausea). The mindfulness module included observing/describing versus judging for staying in the present moment. Examples of observing/describing practices include the following: exercise of eating a raisin and describing the experience; exposure with shake of unknown content and caloric density practicing mindfulness skill. Examples of no judging practices are as follows: focusing on breathing without exerting control, using the finger trap to elicit and remind the experience of greater control inducing more discomfort in order to learn to let it go.

An interoceptive program employed IE exercises to reduce panic, panic-related fears, and general anxiety in individuals with Panic Disorder [110]. It included six sessions of 35% CO_2_ inhalation and individualized activities for the patients, according to what was especially distressing for them, for instance spinning in a chair (dizziness) and running upstairs (heart rate increasing). It drew from a previous study report [111] that showed reduction in autonomic panic sensations, avoidance symptoms, and frequency of panicking with exposure to CO_2_ inhalation and beta-blockers. Two patterns of responses were confirmed: habituation of fear, with decrements in pre- and post-inhalation anxiety, and sensitization of fear, characterized by low levels of pre-inhalation anxiety but steady increases in anxiety following each successive inhalation [110]. Individuals who showed the first pattern response, the habituators, reported lower levels of agoraphobic fears and avoidance than the nonhabituators. The authors speculated that IE sessions served to attenuate fearful thoughts. In addition, exposure may foster emotion regulation, such as cognitive re-evaluation, since there was no chance to escape from uncomfortable interoceptive sensations with a slower process of change in nonhabituators [110].

Finally, a case report showed the efficacy of a repeated application of an acoustic startle stimulus served as a feasible interoceptive exposure strategy for a patient with PTSD with exaggerated startle responses [112]. The treatment included trauma-related imaginal and in vivo exposure with cognitive restructuring, together with psychoeducation, and emotion regulation strategies (e.g., progressive relaxation, grounding). During and after seven trials, the results showed the elicitation of trauma imaginal and memories, with a progressive decrease in general anxiety and distress, as well as improved mood.

## 4. Methodology of Empirical Studies’ Review

In the previous sections, we attempted to offer operational definitions for the constructs of interoceptive ability, emotion regulation, and mind–body interventions through theoretical and empirical articles. In the following section, we are going to explore the impact of mind–body interventions on interoceptive ability through a review of empirical studies (summarized in Table 3 and Table 4).

As we aim to cover both top-down and bottom-up mind–body interventions, we conducted two parallel searches through Pubmed and Psycinfo databases (the first and second author confronted their searches). In the first one, we employed mindfulness programs as a representative case of top-down mind–body interventions. Therefore, we cross-referenced “Interoception” OR “Interoceptive” (a catch-all term for “interoceptive perception” and “interoceptive ability”) AND “Mindfulness” (a term for Mindfulness-based Interventions).

In the second one, we cross-referenced “Interoception” OR “Interoceptive” AND the keywords we chose as representative of bottom-up interventions. First, we used “body movement”, “emotional expression”, and “catharsis”, as they are central to bottom-up interventions as aforementioned according to EDER model. Second, we used “qigong” as representative of a movement-based contemplative practice more oriented towards bottom-up processing. Third, we used “body-psychotherapy” as a keyword for body-oriented therapy based on bottom-up processing. The terms had to be present in any field.

Articles were assessed based on the following criteria: (I) English-written articles published in international peer reviewed journals. English is the language used in the majority of the scientific literature on this subject, even though we are aware that important data can be extracted also from articles written in other languages. (II) Clinical and controlled trials for top-down and for bottom-up: we narrowed down the search to clinical and controlled trials to avoid design bias and obtain more solid evidence on the effect of mind–body interventions. (III) Articles published between January 2010 and September 2023: we selected this time frame as there has been an increasing attention towards the theme here investigated in the last decade.

In the first search, a total of 72 articles were assessed for eligibility. Among these, 51 studies were excluded for the following reasons: duplicate articles; studies without operative definitions of mindfulness-based interventions (e.g., interventions defined under generic names such as meditation or Buddhist meditation); studies that employ mindfulness as trait and not as intervention; studies that do not explicitly measure interoceptive ability; studies that are cross-sectional at baseline of controlled trials; qualitative studies. Finally, 21 studies that met the eligibility criteria described above were included.

From the second search about bottom-up interventions, a total of 24 results for “body movement”, nine results for “emotional expression” and 0 for “catharsis”; three results for “qigong”, 33 results for “body psychotherapy” emerged that eventually were sorted, including just five studies that met the eligibility criteria.

## 5. Results

From the review on empirical studies that include top-down and bottom-up mind–body interventions and their impact on interoception from January 2010 to September 2023, according to our eligibility criteria, a total of 26 empirical studies resulted and are illustrated in Table 3 (top-down interventions) and Table 4 (bottom-up interventions). Except for two cases [47,125] measuring only IAc, all studies measured IS mostly with MAIA. Five studies [113,117,119,126,133] measured both IAc and IS, comparing objective and subjective evaluation. Three studies included the largest number of participants [47,114,129]. All studies included a controlled design, a waitlist, or a comparison with an active control group. The majority of participants are Caucasians living in North America or Europe. One study took place in Brazil [119] and three studies took place in Eastern countries [120,131,133].

Overall, studies showed significant improvements in different subdomains of self-reported IS. Specifically, most studies reported how interventions were associated with significant improvements of the regulative aspects of IS: Attention Regulation and Self-Regulation. These aspects reflect the extent to which subjects focus on their body states in order to regulate their attention and to get familiar with their emotional and motivational states. Besides the regulative aspects, subscales that improved significantly include: Body Listening, Emotional Awareness and Trusting. Other subscales such as Noticing, Not-Distracting, and Not-Worrying did not improve significantly except for a few cases [121,123]. Some studies point to an overall improvement of IS without specifying the subdomains [126,131,132,134,136].

Another important result is that IS improves independently of the intervention duration. In fact, except for an intervention of 10 min that did not result in improved interoceptive ability [113], we found an increase in IS in all other interventions, whose time duration ranged from 3 days [119] for a brief mindfulness training, to 36 weeks [47] for more structured programs such as ReSource Project. This last study examining IAc showed an increase in IAc due to the program of mindfulness delivered in 36 weeks [47].

The second study examining IAc, instead, showed no significant effects in IAc in meditators as assessed with a heartbeat detection task [125]. Even for the studies that measured both IS and IAc the results are controversial. One study reported no significant difference [113], a few studies reported an increase in IS with no significant difference in IAc [119,126,133], and one study [117] showed an opposite trend, that is, a significant positive effect of the intervention on IAc, but no significant change in IS.

While research on interoceptive ability in mind–body interventions has focused on practices based on top-down emotional processing, the domain of bottom-up processing remains under-investigated. Indeed, we sorted only five empirical studies by using the keywords for bottom-up processing mentioned above and including eligibility criteria (see Table 4). Except for one case [133], whereas no significant effects were revealed in IAc nor in IS, these studies show how interventions based on bottom-up processing improve IS. A study interestingly compared the effects on IS of a mindfulness-based intervention, Mindfulness-Oriented Meditation (MOM), with those of Self Body Brushing intervention, which works bottom-up [123]. Whereas for both interventions significant improvements were observed in the subdomains of Noticing, Attention Regulation, and Body Listening, in Self Body Brushing intervention the subscales of Emotional Awareness and Self-Regulation had a higher increase [123].

Furthermore, some studies considered interoceptive ability as a mediator variable since a change in IS in turn was shown to lower levels of anxiety state [120]. MB-BP, a mindfulness-based blood pressure reduction program, was useful for increasing the overall interoceptive sensitivity that affected behavioral changes in physical activity and dietary approaches [121]. Furthermore, in a sample of women with substance use disorder, gaining interoceptive skills through a mindfulness program (MABT, Mindful Awareness in Body-oriented Therapy) significantly improved their emotion regulation of craving [127].

Two studies showed interoceptive sensibility to decrease the difficulties in emotion regulation (measured by the Difficulties in Emotion Regulation Scale DERS) [122,128]. An increased emotion regulation also was revealed to improve, in turn, subjective well-being [123]. This is in line with the improvements reported for the subscale of Emotional Awareness in several studies [114,115,116,119,120,123,130,135] and other information related to emotion regulation. For instance, one study reported significant improvements in both the physiologic (Respiratory Sinus Arrhythmia RSA) and self-report (measured using the DERS) indices of emotion regulation due to a significant change in IS after the intervention [128]. In addition, one study showed higher increase in the subscales of MAIA (Body Listening and Emotional Awareness) after a socio-emotional dyad intervention compared to a mindfulness-based solitary practice [129]. The same study showed an increase in cognitive reappraisal, as a strategy of emotion regulation, mediated by the increased subdimension of IS, Self-Regulation.

## 6. Discussion

In the first part of the article, we addressed the constructs of interoceptive ability, including epistemological and methodological aspects, and mind–body interventions in relation to emotion processing and regulation. Mind–body interventions operate on the brain–body axis by initiating from mental processing at the level of the cerebral cortex (top-down) or afferent pathways from the periphery to the brainstem and cerebral cortex (bottom-up). We started from the premise that these bidirectional processes also define ways of experiencing and regulating emotions, considering the link between interoceptive ability and emotion regulation as a prominent research subject in this context. At the end of the first part of the review, we illustrated some insights from case studies about interoceptive exercises and in vivo exposure, especially carried out with individuals with Panic Disorder, Eating disorder, and PTSD. Interoceptive exercises based on active movement and breathing practices (e.g., hyperventilation), were often combined with teaching of some mindfulness skills to resist to the distress generated by interoceptive sensations (e.g., pressure in stomach, nausea) and with psychotherapies as CBT. This combination was shown to reduce the clinical symptoms, such as anxiety, phobias, and avoidance behaviors. According to the authors, the reason why these interventions work can be understandable considering the physiological phenomenon of habituation that decreases the arousal reaction over time and considering the emotion regulation processes affected by interoception. Indeed, in line with previous interpretations about the effectiveness of interoceptive exercises (110), we could hypothesize that the interoceptive intervention lowers the normal defense mechanisms, since there is no chance to escape from uncomfortable interoceptive sensations, and allows a cognitive re-evaluation of the emotional experience, attenuating the fearful thoughts. Hence, a close relationship between interoception and emotion regulation processes was revealed in the case studies.

In the second part of the review, we explored the impact of mind–body interventions on interoceptive ability through a review of empirical studies (Randomized or NoRandomized Control Studies and Clinical Studies) from 2010 to 2023.

As a first result, empirical studies revealed that interoceptive ability is a key-aspect of practices based on top-down processing such as mindfulness-based interventions. Yet, whether and how practices based primarily on bottom-up processing, such as those involved in spontaneous body movement and emotional expression, may impact the capacity to sense the physiological condition of the body is still under-investigated. This limitation is partly due to the widespread reliance on the CER approach to emotion regulation based on appraisal theory, which takes emotion regulation as a process that unfolds over time, but which fails to account for those practices that rely on spontaneous body movement and emotional expression. In this regard, the EDER approach needs to be integrated in the study of interoception.

Converging evidence from empirical studies, mostly clinical trials, indicate how mind–body interventions enhance interoceptive ability, here defined as the capacity to detect, interpret, and consciously integrate signals related to the physiological condition of the body, which is intrinsically related to emotion regulation. Specifically, the results point to a correlation between mindfulness-based interventions and IS, with the subscales of Attention Regulation, Self-Regulation (the regulative aspects of IS), Body Listening, Emotional Awareness, and Trusting particularly affected by the training. Therefore, they show that mindfulness improves the attention to body sensations, the capacity to listen to the body for insight and regulative purposes, and the awareness of the connection between body sensations and emotional states. Additionally, it allows experiencing body signals as safe and trustworthy. If we relate these findings with the studies reported in Table S1, regarding interoception and emotion, there is evidence to contend that interoceptive ability is a crucial aspect associated with the effects of mind–body interventions on emotion regulation. For instance, it has been shown how the perception of visceral signals, the awareness of emotional states, and their regulation activate the same brain structures [38,41,42].

Bottom-up interventions seem to be linked to overall improvements of IS, even though in this regard there is still limited information. Yet, important data emerge from a study that compared a top-down intervention (MOM) with a bottom-up intervention (SBB). Whereas significant changes were observed in both groups in the scales of Noticing, Attention Regulation, and Body Listening, the SBB group reported higher points in the scales of Emotional Awareness and Self-Regulation. In other words, the bottom-up intervention helped participants to better recognize and regulate their body sensations associated with emotional states. This result tells us that, although bottom-up interventions do not focus on attentional processes, the stimulation of the sensorimotor system (including proprioceptive changes) triggers involuntary attention to the physiological condition of the body (see Figure 2b). Moreover, the perception of arising sensations may help participants to familiarize themselves with their emotional states and use this information to manage emotionally charged situations, having a regulative function according to the EDER model.

In general, given the variability of the subscales among all the cases here examined, it is assumed that, besides the regulative aspects, different interventions affect different dimensions of interoceptive ability, which could be beneficial if integrated. In light of this, studies on interoceptive ability emphasize the need to delve deeper into the potential clinical utility of integrated mind–body interventions, especially combining top-down and bottom-up programs.

We also found a discrepancy of findings between IAc and IS. The majority of studies that measured both dimensions reported an increase in IS with no significant difference in IAc. This result could be explained by the fact that participants are more confident in the perception of their internal body states, but that such confidence does not reflect an actual accuracy when measured in behavioral performances. Interestingly, in two studies [117] the intervention (MBBS) resulted in a significant change in IAc, but no significant change in IS. Considering that in these cases IS was quantified using a combination of the participants’ self-rated confidence and the subscale of the German EDI-2 questionnaire, the result could be correlated to the use of questionnaires other than MAIA. At the same time, improved IAc is not in line with the majority of studies included in the table, which report no significant changes in this dimension regardless of the intervention time length.

The discrepancy between IAc and IS may alternatively be explained by the fact that, although scholars focused on cardiac afferent signaling to measure accuracy, there are different sensory channels of interoception that could be investigated such as the respiratory and gastrointestinal axes [13]. Hence, to measure IAc with heartbeat counting task could be considered a methodological limitation according to the results of our review. Indeed, the validity of the heartbeat counting task has been questioned and a weak correspondence between measures of cardiac and respiratory IAc has been reported [30]. Given the centrality of breathing to mindfulness-based practices, it is likely to hypothesize that training could improve accuracy of the signals in the respiratory axis more than the cardiac one. To overcome this limitation, experimental tasks that focus on the respiratory or gastrointestinal level could be further employed. Likewise, future research could investigate how the integration of sensor-equipped technological devices, which offer immediate feedback on physiological signals like heart rate and skin conductance, may empower individuals to grasp their internal states and offer additional measures at the same time [137].

A second limitation emerging from the results of our review is given by the difficulty in providing an operative definition and integrative framework for the constructs here examined. With regard to interoceptive ability, in this article we referred to the multidimensional model proposed by Garfinkel et al. [26] and recently extended to include two additional dimensions [31]. Yet, the literature review reveals how studies on interoception are built on different conceptualizations of interoceptive ability or take into consideration only one dimension among the ones here examined. This poses an epistemological problem to research in this field.

As for mind–body interventions, although the scientific literature is increasingly paying attention to them, we are aware that articulating an integrative framework for such a wide construct poses important challenges. These are due to the variety of practices and methods that impact the bidirectional pathway between the brain and the body, and, more importantly, to the variety of contexts in which they may be proposed. In fact, there is a distinction between mind–body interventions applied in programs for the promotion of mental health and well-being, and interventions based predominantly on emotional release that require a therapeutic setting. A further limitation is given by a preponderance of English-language sources in the scientific literature. Considering that several mind–body interventions come from Non-Western cultures, English descriptions risk neglecting important information and meanings.

In light of the observations and limitations above, future studies could be improved by the following recommendations. First of all, it would be advisable to investigate the relation between interoceptive ability and emotion regulation in embodied practices based on spontaneous body movement and emotional expression. Knowing how important psychological factors are to the quality of life and well-being of individuals, mind–body psychotherapies that employ bottom-up processing as a therapeutic means should promote systematic RCT studies, for instance clinical retrospective studies [138,139,140]. Also, more research is needed to explore the psychophysiological correlation between spontaneous body movement and emotional expression, considering the valid assumption that information processing begins in the sensorimotor periphery. To this aim, the enactive approach to emotion [66], and more generally the paradigm of embodiment needs to be assimilated into empirical research [67,68]. From this view, cognition is not something that happens solely in the brain but is distributed throughout the entire body and its interaction with the environment. The perspective of our study resonates with the principles of grounded cognition, which asserts that cognitive processes are rooted in bodily experiences and sensorimotor interactions [141,142].

Obviously, we are aware that the CNS plays a pivotal role, because it is composed of several areas that are connected between each other and that create large-scale networks, whose activity is associated with the flow of consciousness that characterizes us moment by moment [143]. Hence, in this view, interventions could integrate innovative non-invasive brain stimulation techniques like transcranial direct current stimulation (tDCS), which has already been employed in a wide variety of clinical populations (refer to Orrù et al. [144,145,146]) to enhance clinical outcomes. In the context of interoceptive ability, the goal may be to utilize these methods to augment or regulate interoception by stimulating deep cerebral areas like the insula [147].

Finally, it is advisable that operative definitions of interoceptive ability and mind–body interventions consider the first-person perspective. For interoceptive ability, it is suggested the inclusion of affective consciousness and the significance of qualitative and phenomenological methods of inquiry beyond quantitative methods. As for mind–body interventions, it is important to develop a more integrative framework that takes into consideration their biological, psychological, and sociocultural aspects in an interdisciplinary way. In this regard, research could benefit from confronting what we already know about top-down and bottom-up emotional processing with qualitative and ethnographic studies focused on local or indigenous phenomenologies of interoception, including non-English sources. In addition, other criteria of classification underlined above such as modalities of attention, type of movement, and physical contact could be assessed together with interoception, in order to provide a more integrated definition of mind–body interventions.

## 7. Conclusions

This review has shown converging evidence pointing to the positive effect of both top-down and bottom-up interventions on the capacities of emotion regulation through the mediating effect of interoceptive ability. In sum, we contend that interoceptive ability is a crucial aspect associated with the effects of mind–body interventions on emotion regulation. Additionally, we suggest that if studied through quantitative and qualitative methods, interoceptive ability may serve as a general construct that allows a more integrated view of the polarities related to the spectrum of embodied experience: top-down and bottom-up emotion processing, observational and non-observational body awareness [23], conscious and unconscious levels of interoception [12].

## Figures and Tables

**Table 1:** Interoceptive Ability’s dimensions based on a Multidimensional Framework [26,31].

Dimension	Definition	Example of Measurement
Accuracy (IAc)	Grade of accuracy in perceiving one’s body state. It is the core element of interoception since it aims at objectively quantifying individual differences in behavioral performances	Heartbeat Detection Task [27,28].
Sensibility (IS)	Subjective measure that reflects both individuals’ beliefs in their interoceptive ability and confidence in their performance accuracy on an experimental task	Self-report Questionnaires (Body Perception Questionnaire—BPQ [32]; Multidimensional Assessment of Interoceptive Awareness—MAIA [33])
Awareness (IA)	Metacognitive awareness that refers to the correspondence between interoceptive accuracy and sensibility	Receiver operating characteristic curve analysis to quantify the extent to which confidence predicts accuracy
Attention	The mode of attention through which interoceptive signals reach conscious awareness	MAIA [33]; Qualitative Methods (e.g., in-depth interviews, semi-structured and unstructured interviews)
Attribution of signals	The specific attribution and interpretation of interoceptive signals, which may be influenced by the dispositional style, affect, and sociocultural context	Qualitative Methods (e.g., in-depth interviews, semi-structured and unstructured interviews)

**Table 2:** Mind–Body Interventions’ Criteria according to Top-Down/Bottom-Up Processing.

	Top-Down	Bottom-Up
Attentional Style	Voluntary attention (e.g., focused attention, open monitoring)	Involuntary attention
Movement	Voluntary movement; imagined movement	Spontaneous and involuntary movement
Administration	Self-administered or Delivered by another person	Self-administered or Delivered by another person
Physical Contact	Self-contact may be included	Relational physical contact or touch may be included
Context	Psychotherapy Recreational context Spiritual and healing rituals	Psychotherapy Recreational context Spiritual and healing rituals

**Table 3:** Selected Empirical Studies on the Effects of Mind–Body Interventions on Interoceptive Ability searched with “Mindfulness-based Interventions” for Top-down Processing.

Authors	Study Design	Mind–Body Intervention	Duration	Sample Size	Dimensions Measured	Impact on Interoceptive Ability
Aaron et al. [113]	RCT with control group (listening to a reading from a textbook)	MBBS	10 min	N = 76 (50 female, M age = 19.70). Healthy young adults in North America	IAc (assessed using a heartbeat detection task) IS (quantified as participants’ self-rated confidence in their accuracy detecting heart beats)	↔ IAc ↔ IS
Bornemann and Singer [47]	RCT (3 training cohorts + a control cohort)	ReSource Project	36 weeks	N = 332 (187 female, M age = 40.8). Healthy adults in Germany	IAc (assessed using a heartbeat detection task)	↑ IAc
Bornemann et al. [114]	RCT with a retest control group	MBBS and Breath Meditation	12 weeks	N = 232 (127 female, M age = 42.45). Healthy adults recruited from different German cities	IS (MAIA)	↑ Attention Regulation, Emotional Awareness, Self-Regulation, Body Listening Trusting ↔Noticing, Not-Distracting, Not-Worrying
deJong et al. [115]	RCT with a control group (waitlisted)	MBCT	8 weeks	N = 40 (30 female). Adults with chronic pain and comorbid active depression, recruited from outpatient clinics in North America	IS (MAIA)	↑ Emotional Awareness, Self-Regulation ↔Noticing, Not-Distracting, Not-Worrying, Attention Regulation, Body Listening, Trusting
Fazia et al. [116]	RCT with a control group (waitlisted)	Mindfulness-Based Meditation Training	12 weeks	N = 42 (29 female, M age = 34.1). Healthy adult employers of a consulting company located in Italy	IS (MAIA)	↑ Emotional Awareness, Self-Regulation, Body Listening, Trusting ↔Noticing, Not-Distracting, Not-Worrying, Attention Regulation
Fischer et al. [117]	We considered 2 studies. Study 1: RCT with an active control group (listening to an audio book) Study 2: RCT with a control group	MBBS	8 weeks	Study 1: N = 50 (39 female, M age = 22.5). Study 2: N = 36 (18 female; M age = 22.5). Healthy students recruited in Germany	IAc (assessed using a heartbeat detection task); IS (quantified as participants’ self-rated confidence in their accuracy detecting heart beats + subscale “interoceptive awareness” from the German EDI-2 questionnaire)	Study 1 e 2: ↑ IAc ↔ IS
Fissler et al. [118]	RCT with an active control group	MBCT	8 weeks	N = 74 (42 female, M age = 17.3). Depressed patients recruited in Germany	IS (MAIA)	↑ Attention Regulation, Self-Regulation, Body Listening, Trusting ↔Noticing, Not-Distracting, Not-Worrying, Emotional Awareness
Lima-Araujo et al. [119]	RCT with an active control group (listening to audio on educational health)	Brief mindfulness training	3 days	N = 40 (20 female; M age 24.15). Healthy students from Natal, Brazil	IAc (assessed using a heartbeat detection task); IS (MAIA)	↔ IAc ↑ Attention Regulation, Emotional Awareness, Self-Regulation, Body Listening, Trusting ↔Noticing, Not-Distracting, Not-Worrying,
Lin and Yeh [120]	RCT with a control group (receiving usual medical care)	Mindful Walking	8 weeks	N = 78 (2 female; M age = 72.21). Patients with chronic obstructive pulmonary disease recruited from the outpatient departments of a medical center in northern Taiwan	IS (MAIA only 5 subscales)	↑ Emotional Awareness ↔ Noticing, Attention Regulation, Self-Regulation, Body Listening
Loucks et al. [121]	Stage 1 clinical trial with one year follow-up	MB-BP	10 weeks	N = 48 (29 female; M age 60). 60% American adults assessed with hypertension risk factors	IS (MAIA)	↑ Noticing, Not-Worrying, Attention Regulation, Self-Regulation, Body Listening, Trusting ↔ Not-Distracting, Emotional Awareness
Loucks et al. [122]	Stage 1 RCT with a control group (offered a referral to counseling services upon request)	MB-College	9 weeks	N = 96 (65 female; M age = 20). Healthy American university students	IS (MAIA)	↑ Attention Regulation, Self-Regulation ↔ Noticing, Not-Distracting, Not-Worrying, Emotional Awareness, Body Listening, Trusting
Matiz et al. [123]	Non-Randomized Pilot Study (2 training groups with a control group)	SBB versus MOM	4 weeks 8 weeks	N = 49 (34 female; M age = 42.65). Healthy adults from Northern Italy	IS (MAIA)	↑ Noticing, Attention Regulation, Body Listening for both groups ↑ Emotional Awareness, Self-Regulation for the SBB group
Mehling et al. [124]	RCT with a control group (waitlisted)	Integrative Exercises versus MBSR	12 weeks	N = 47 (9 female, M age 46.8). American war veterans with PTSD	IS (MAIA)	↑ Self-Regulation
Melloni et al. [125]	RCT (long term and short term meditators with a control group of non-meditators)	MBSR	8 weeks	N = 9 completed the MBSR program (short term meditators); N = 10 long-term practitioners (mean 4.5 years) and N = 10 non-meditators. Healthy adults.	IAc (assessed using a heartbeat detection task)	↔ IAc
Parkin et al. [126]	Study 3: RCT with a control group	MBSR MBCT	8 weeks	Study 3: N = 19 (15 female; M age = 46.94). Healthy adults recruited in Germany	IAc (assessed using a heartbeat detection task) IS (quantified as participants’ self-rated confidence in their accuracy detecting heartbeats)	↔ IAc ↑ IS
Price et al. [127]	RCT 3 groups: usual medical treatment + MABT (intervention); usual medical treatment + educational program; usual medical treatment	MABT	12 weeks	N = 187 (M age 35.3). Adult women in intensive outpatient treatment for substance use disorder at community clinics in the Pacific Northwest of the United States	IS (MAIA)	↑ IS (subscales not specified)
Roberts et al. [128]	RCT with a control group (receiving therapeutic support) 3-month follow-up	MORE	8 weeks	N = 96 (65 female; M age = 20). Adult with chronic pain recruited from primary care and pain clinics in Utah, USA	IS (MAIA)	↑ Self-Regulation ↔Noticing, Not-Distracting, Not-Worrying, Attention Regulation, Emotional Awareness, Body Listening, Trusting
Silveira et al. [129]	RCT (2 intervention groups with a control group)	MB Solitary Practice versus SE	10 weeks	N = 285 Adults with emotion processing deficits of alexithymia from a mental health project conducted in Berlin, during the COVID-19	IS (MAIA)	↑ Attention Regulation, Self-Regulation, Body Listening for the SE group ↔Noticing, Not-Distracting, Not-Worrying, Attention Regulation, Emotional Awareness, Trusting
Thomas et al. [130]	Stage 1 RCT with an active control group (exercise and nutrition counseling)	MORE	10 weeks	N = 51 (M age = 57.92). Women overweight and obese cancer survivors	IS (MAIA)	↑Attention Regulation, Emotional Awareness Self-Regulation, Body Listening, Trusting ↔Noticing, Not-Distracting, Not-Worrying
Zhang et al. [131]	RCT with an active control group	MBCP	9 weeks	N = 183 (M age = 32.5). Pregnant women in Hong Kong	IS (MAIA)	↑ IS (subscales not specified)

Note: ↑ = the variables improved and were statistically significant from baseline or from the control group; ↔ = no or non-statistically significant difference from baseline or from the control group.

**Table 4:** Selected Empirical Studies on the Effects of Mind–Body Interventions on Interoceptive Ability searched with “body movement”, “emotional expression”, “catharsis”, “qigong”, and “body-psychotherapy” for bottom-up processing.

Authors	Study Design	Mind–Body Interventions	Duration	Sample Size	Dimensions Measured	Impact on Interoceptive Ability
Ahmadi [132]	RCT with an active control group (core stability exercises)	Feldenkrais	5 weeks	N = 59 (M age 40.7). Patients with chronic low back pain recruited from an Iranian University	IS (MAIA)	↑ IS (subscales not specified) Feldenkrais group versus Back School Group at the follow-up stage
Chang et al. [133]	RCT with a control group (receiving medical routine care)	Chan-Chuang Qigong	15 weeks	N = 60. Women patients with breast cancer during chemotherapy	IAc (assessed using a heartbeat detection task) IS (MAIA)	↔ IAc ↑ Self-Regulation ↔Noticing, Not-Distracting, Not-Worrying, Attention Regulation, Emotional Awareness, Body Listening, Trusting
Matko et al. [134]	Single Case Multiple Baseline Design	Yoga	8 weeks	N = 42 (35 female, M age = 26.62). Healthy adults from a German community	IS (measured as “body awareness” through a questionnaire partly developed from MAIA)	↑ IS
Paolucci et al. [135]	RCT with an active control group (receveing a rehabilitating program)	Feldenkrais versus Back School Program	5 weeks	N = 53 (44 female, M age = 60.95). Adults with chronic low back pain from an outpatient rehabilitation center of a hospital in Rome, Italy	IS (MAIA)	↑ IS (all subscales) for both groups ↑ Emotional Awareness for the Feldenkrais group versus Back School Group at the follow-up stage
Payne et al. [136]	Non-Randomized Pilot Study	A set of Qigong exercises	16–20 weeks	N = 26 (M age = 67.3). Healthy flight attendants (all females) recruited from the Northeastern region of the US	IS (MAIA)	↑ IS (subscales not specified)

Note: ↑ = the variables improved and were statistically significant from baseline or from the control group; ↔ = no or non-statistically significant difference from baseline or from the control group.

## Supplementary Materials

The following supporting information can be downloaded at: https://www.mdpi.com/article/10.3390/bs14111107/s1.

## Glossary

MBBS	(Mindfulness-based body scan): a central element of MBSR where attention is paid to different parts of the body, sensations, heartbeat, and breathing in the present moment without any judgment or criticism of the moment to moment experience
ReSource	a Mindfulness-Based Intervention composed of three modules: Presence (breathing meditation and body scan), Affect (Loving-kindness meditation and a socio-affective dyadic exercise), Perspective (Observing-thoughts meditation and a socio-cognitive dyadic exercise).
MBCT	(Mindfulness-based cognitive therapy): an approach to psychotherapy that combines cognitive behavioral therapy (CBT) with mindfulness meditative practices.
MB-BP	(Mindfulness-Based Blood Pressure Reduction): a training adapted from MBSR to direct participants’ mindfulness skills towards modifiable determinants of blood pressure.
MB-College	a program based on MBSR that aims at young adult well-being through education and biofeedback, including specific modules focused on awareness of diet, physical activity, alcohol use, stress, sleep, social relationships, social support, and performance.
SBB	(Self Body Brushing).
MOM	(Mindfulness-Oriented Meditation).
Integrative Exercises	These include a dynamic warm-up (e.g., jumping, walking, running), a series of aerobic (e.g., running, steps, lunges), resistance training exercises, and yoga postures.
MBSR	(Mindfulness-based stress reduction): an eight-week evidence-based program designed to assist people with stress, anxiety, depression and pain. It combines meditation, body awareness, yoga and exploration of patterns of behavior, thinking, feeling and action.
MABT	(Mindful Awareness in Body-oriented Therapy): a therapeutic approach that trains interoceptive awareness skills to promote emotion regulation and well-being using the combination of manual, mindfulness, and psychoeducational approaches.
MORE	(Mindfulness-Oriented Recovery Enhancement): an integrative behavioral intervention designed to target mechanisms underlying appetite dysregulation; it combines traditional mindfulness training with cognitive reappraisal and strategies designed to reverse the allostatic shift in reward salience.
MB Solitary Practice	A mindfulness-based program performed daily on a smartphone app and complemented by weekly coaching sessions
SE	(Socio-Emotional Affect Dyad)
MBCP	(Mindfulness-Based Childbirth and Parenting)
FELDENKRAIS	A method based on awareness through movement lessons, which are verbally guided explorations of movement.
BACK SCHOOL PROGRAM	A rehabilitative program based on explanations and exercises administered by physiotherapists.
