# Supplementary material for: Interoceptive Ability and Emotion Regulation in Mind–Body Interventions: An Integrative Review
Source: Behav Sci (Basel). 2024 Nov 18;14(11):1107. doi: 10.3390/bs14111107 (PMC11591285; doi:10.3390/bs14111107)
Supplement: Supplementary file 1 [file behavsci-14-01107-s001.zip › behavsci-3205876-supplementary.pdf]

Table S1. Relationship between Interoceptive Ability and Emotion

Empirical studies informative of the relationship between interoceptive ability (measured through the dimension of IAc) and emotion regulation. These studies were drawn from the bibliography of the research articles selected in the review (see Table 3,4).

**Authors**	**Objective**	**Results**	**Relationship with Emotion and Emotion Regulation**
Bornemann & Singer [47]	Investigate whether contemplative mental practice can increase attentional and interoceptive ability (IAc), socio-affective, and socio-cognitive abilities	Changes in heartbeat perception task were concomitant with and predictive of changes in emotional awareness	IAc is positively correlated with improvements in emotional awareness
Critchley et al. [5]	Measure regional brain activity by fMRI during an interoceptive task wherein subjects engaged in a heartbeat detection task	Enhanced activity in insula, somatomotor and cingulate cortices	The right anterior insula supports a representation of visceral responses accessible to awareness, providing a substrate for subjective feeling states
Ernst et al. [40]	Evaluate how interoceptive awareness impacts and modulates neural activity during empathy using fMRI	Interoceptive awareness significantly enhanced neural activity during empathy in bilateral anterior insula and other cortical midline regions	Interoceptive processing modulates emotion-related neural activity
Füstös et al. [43]	Investigate the neural dynamics of reappraisal of emotional responses of participants who differed with respect to IAc	Reappraisal was accompanied by reduced arousal and significant modulation of late neural responses	IAc facilitates the downregulation of affect-related arousal when applying reappraisal as emotion regulation strategy
Herbert et al. [38]	Examine the relationship between IAc and both the subjective emotional experience and the processing of emotional pictures	The heartbeat perception score correlated significantly positive both with visually evoked potentials (assessed by EEG analysis) and slow wave amplitudes as well as with the arousal ratings for emotional pictures	There is a significant and strong association between IAc and the intensity of emotional experience as well as the processing of emotional stimuli

Kever et al. [45]	Investigate whether individual differences in IAc are associated with the habitual use of two main emotion regulation strategies: reappraisal and suppression	Individuals with higher IAc showed both greater habitual reappraisal and suppression use compared to those with lower IAc	IAc facilitates the selection and implementation of antecedent-focused as well response-focused emotion regulation strategies
Pollatos et al. [42]	Investigate the relationship between interoceptive ability (IAc) and emotion-related brain activity	Heartbeat perception scores correlated significantly and positively with both the mean arousal rating as well as with the mean amplitudes in the visually evoked potentials (from EEG analysis) and slow wave amplitudes	Strong relationship between IAc and the cortical processing of emotional stimuli
Pollatos et al. [46]	Explore whether IAc fosters coping with social exclusion and flexibility in emotion regulation	Study 1 revealed that the effects of ostracism have less impact on participants with stronger IAc. Study 2 revealed that IAc was associated with habitually more efficient emotion regulation strategies	IAc helps reducing aversive states provoked by social exclusion, probably due to the fact that IAc is associated with emotion regulation strategies
Weiss et al. [44]	Examine the interaction of interoceptive ability (IAc) and self-regulation in somatoform patients	IAc was associated with better self-regulation capacities	IAc is positively associated with frustration tolerance, affect differentiation and affect tolerance
Wiens et al. [41]	Investigate the relationship between interoceptive ability (IAc) and affective responses to film clips targeting different emotional valances	Good heartbeat detectors reported more intense emotions than poor detectors across 3 emotional valances (amusement, anger, fear)	IAc plays a role in the experience of the intensity of emotions
Zaki et al. [39]	Examine the neural convergence of interoception and emotional experience through heartbeat monitoring while participants rated their own emotion	A circumscribed area, spanning insular cortex and adjacent inferior frontal operculum, was engaged during both heartbeat and emotion monitoring	Second-order representations (elaborated mainly in anterior insula) are a key feature of emotion generation and experience

Table S2. Examples of Case Studies on Interoceptive Interventions

Authors	Program	Disorder	Effects
Ball & Otto [107]	11-13 sessions of interoceptive and in vivo exposure (IE) + CBT	Eating disorder	↑ weight gain<br>↓ reduced choking phobia
Boswell et al. [108]	4 sessions of IE (e.g. hyperventilation; thin straw breathing; spinning in place; gulping water until full; smelling high calorie food; wearing tight cloth) +CBT	Eating disorder	↓ interoceptive deficits and anxiety
Plasencia et al. [109]	6 sessions of acceptance-based interoceptive exposure (ABIE); IE + Mindfulness practices of Observing and Describing	Eating disorder	↑ weight gain<br>↓ anxiety symptoms.
Beck, Shipherd, & Zebb [110]	6 sessions of 35% CO_2 inhalation, and individualized activities (e.g. spinning in a chair, running up stairs)	Panic Disorder	↓ panic, panic-related fears, and general anxiety<br>↓ levels of agoraphobic fears and avoidance
Griez & van den Hout [111]	6 sessions of CO_2 and propranolol inhalation + beta-blockers	Panic Disorder	↑ state anxiety and agoraphobic anxiety<br>↓ autonomic panic sensations, avoidance symptoms, and frequency of panicking
Asmundson & Carleton [112]	7 sessions trauma-related imaginal and in vivo exposure + cognitive restructuring + psychoeducation + emotion regulation strategies (e.g. progressive relaxation, grounding) + repeated application of an acoustic startle stimulus served as a feasible interoceptive exposure strategy	PTSD with exaggerated startle	↑ trauma imaginal and memories<br>↓ general anxiety and distress

Note:

↑= the variables improved and were statistically significant from baseline

↔= no or non-statistically significant difference from baseline

↓=the variables declined and were statistically significant from baseline
